# Analysis of 61 SNPs from the CAD specific genomic loci reveals unique set of SNPs as significant markers in the Southern Indian population of Hyderabad

**DOI:** 10.1186/s12872-022-02562-4

**Published:** 2022-04-05

**Authors:** Manjula Gorre, Pranavchand Rayabarapu, Sriteja Reddy Battini, Kumuda Irgam, Mohan Reddy Battini

**Affiliations:** 1 Department of Genetics and Biotechnology, Osmania University, Hyderabad, 500007 India; 2 Molecular Anthropology Laboratory, Indian Statistical Institute, Hyderabad, India; 3 Dr Pinnamaneni Siddhartha Institute of Medical Sciences and Research Foundation, Vijayawada, India

**Keywords:** Coronary artery disease, GWAS, 11q23.3, 9p21.3, SNPs, Indian population

## Abstract

**Background:**

The present study is a part of the major project on coronary artery disease (CAD) carried out at Indian Statistical Institute, Hyderabad to investigate the pattern of association of SNPs selected from the CAD specific genomic loci. The study is expected to portray the genetic susceptibility profile of CAD specifically in the Southern Indian population of Hyderabad.

**Methods:**

The study was conducted in a cohort of 830 subjects comprising 350 CAD cases and 480 controls from Hyderabad. A prioritized set of 61 SNPs selected from the NHGRI GWAS catalogue were genotyped using FluidigmNanofluidic SNP Genotyping System and appropriate statistical analyses were used in interpreting the results.

**Results:**

After data pruning, out of 45 SNPs qualified for the association analysis, four SNPs were found to be highly significantly associated with increased risk for CAD even after Bonferroni correction for multiple testing (*p* < 0.001). These results were also replicated in the random subsets of the pooled cohort (70, 50 and 30%) suggesting internal consistency. The ROC analysis of the risk scores of the significant SNPs suggested highly significant area under curve (AUC = 0.749; *p* < 0.0001) implying predictive utility of these risk variants.

**Conclusions:**

The *rs10455872* of *LP(A)* gene in particular showed profound risk for CAD (OR 35.9; CI 16.7–77.2) in this regional Indian population. The other significant SNP associations observed with respect to the pooled CAD cohort and in different anatomical and phenotypic severity categories reflected on the role of genetic heterogeneity in the clinical heterogeneity of CAD. The SNP *rs7582720* of *WDR12* gene, albeit not individually associated with CAD, was found to be conferring significant risk through epistatic interaction with two SNPs (*rs6589566, rs1263163* in *ZPR1*, *APOA5-APOA4* genes) of the 11q23.3 region.

**Supplementary Information:**

The online version contains supplementary material available at 10.1186/s12872-022-02562-4.

## Background

Coronary artery disease (CAD) is a multifactorial disorder involving both genetic and environmental factors and is characterized by genetic as well as etiologic heterogeneity. Hence, identifying the causative factors for CAD development and/or progression is always challenging. The candidate gene approaches have identified approximately 300 genes that belong to a wide range of metabolic pathways to be associated with CAD. The genome wide association studies (GWAS) revealed significant association of 32 chromosomal loci previously [1, 2] whereas, a recent study identified 64 novel genetic loci in the CAD genetic architecture [3]. There were many replication studies on the association of variants at these GWAS loci with CAD in specific populations and meta-analyses which identified the causal genes for CAD development. However, despite the lack of consistency in the association patterns of these genes/loci across the populations, these studies suggested 11q23.3 and 9p21.3 chromosomal regions as the most replicated CAD associated loci across the globe. The candidate gene and GWAS studies revealed that 11q23.3 Apolipoprotein gene cluster region is associated specifically with lipoprotein metabolism which if defective is shown to play an important role in the process of atherosclerosis, the primary event in CAD development [4, 5]. Moreover, 9p21.3 and 11q23.3 loci were shown to have pleiotropic effects harbouring statistically significant Single Nucleotide Polymorphisms (SNPs) associated with various complex disorders including CAD [6]. Given the prominent association of these two loci with CAD, as part of the major project, we have earlier investigated the patterns of association of 95 SNPs of 11q23.3 [7, 8] and 35 SNPs of 9p21.3 [9] chromosomal regions in the Southern Indian population of Hyderabad almost saturating these two genomic regions. We observed rs7865618 of 9p21.3 locus and 12 SNPs from the 11q23.3 region to be significantly associated with CAD and the results have suggested unique patterns of association of different SNPs of these loci with different anatomical and phenotypic categories of CAD and epistatic interactions between SNPs of the same and/or different chromosomal regions [7–9]. Given the significant findings of our previous studies, the present study in the same cohort was focused on the analysis of 61 more SNPs reported with greater than genome wide threshold p value in 3 metabolic syndrome GWAS selected from the National Human Genome Research Institute (NHGRI) GWAS catalogue. We presented here the results on the pattern of association of 61 SNPs with the pathogenesis of CAD, and also of the epistatic interactions between these SNPs and the previously analyzed SNPs of 11q23.3 and 9p21.3 regions. The study might help in comprehensive portrayal of the genetic susceptibility profile of the population of Hyderabad for this important and life-threatening disease.

The population of Hyderabad is a conglomeration of people from different parts of the undivided state of Andhra Pradesh and the mother tongue of most of its population is Telugu, one of the four Dravidian languages. It would be pertinent to note that, despite the subdivision of Telugu population into a number of traditionally endogamous castes and sub castes, Reddy et al. [10] observed genetic differentiation among the populations of Andhra Pradesh to be very low and insignificant. The Markov chain Monte Carlo analysis of population structure which implements model based clustering method for grouping individuals into populations [11, 12] did not reveal any unique population clusters suggesting high degree of genetic homogeneity. This would preclude the possibility of the effect of substructure in the study cohort.

## Methods

### Ethics statement

The study protocol was approved by the Indian Statistical Institute (ISI) Review Committee for Protection of Research Risks to Humans and all experiments were performed in accordance with the relevant guidelines and regulations. Written informed consent was obtained from all the participants as per the guidelines.

### Study design and population

For this case–control study, a total of 1024 subjects comprising 508 CAD cases and 516 controls broadly representing the populations of the undivided Andhra Pradesh were included. The CAD cases were recruited from the CARE hospitals, Hyderabad after their evaluation by interventional cardiologists. Patients with characteristic symptoms of stable/unstable angina pectoris along with varying degrees (generally > 40%) of stenosis in at least one of the major coronary arteries as determined through angiogram were included. Cases with monogenic diseases, valvular heart disease, cardiomyopathy, renal disease, acute and chronic viral or bacterial infections, asthma, tumors or connective tissue diseases, other vascular diseases and familial cases of CAD were excluded from the study. The baseline characteristics of CAD patients recruited for the present study were furnished in a previous publication [7].

The control samples were collected from Hyderabad and its vicinity broadly representing similar ethnic composition, socioeconomic backgrounds as that of the cases and aged above 45 years. The individuals with characteristic features of any of the above-mentioned exclusion criteria and/or with positive family history for the same were not included as part of the controls.

### Data and blood sample collection

The epidemiological and clinical data pertaining to the individuals, who participated in the study, were obtained through personal interviews using a detailed questionnaire and from the hospital records. About 5 ml of peripheral fasting blood sample was collected from each of the subjects by certified medical lab technicians.

### DNA isolation and genotyping

All the blood samples were used for isolation of DNA using phenol chloroform method [13]. The quality and quantity of isolated DNAs were determined with the help of Thermo Scientific Varioskan^TM^Flash Multimode Reader using Quant-iT^TM^PicoGreen® dsDNA Assay Kit. Quantification of the samples was done at Sandor Life sciences, a medical laboratory in Hyderabad. Genotyping was performed for the prioritized set of 61 SNPs, selected from the CAD associated SNPs with greater than genome wide threshold *p* value that were reported in 3 metabolic syndrome GWAS from NHGRI GWAS catalogue, using FluidigmNanofluidic SNP Genotyping System at the same laboratory. Integrated fluidic circuit (IFC) chips were utilized for genotyping which were thermal cycled and, the end-point fluorescent values were measured on Biomark™ system. Final sample wise genotype calls were obtained using Fluidigm SNP Genotyping Analysis software. All the 61 SNPs used for the present study were characterized for genomic localization, function or nearby gene information (Additional file 2: Table S1). Genotype call rate of ≥ 99% was achieved for all the SNPs in 350 cases and 480 controls which were used for the association analyses.

### The anatomic and phenotypic severity categorization of CAD cases

The CAD cases were categorized into four ‘anatomical’ subtypes [8]; (1) cases with 40–70% stenosis and symptomatic for CAD with characteristic atherosclerotic lesions as ‘insignificant’ disease, (2) with > 70% stenosis in any one of the major coronary blood vessel as ‘Single Vessel Disease’ (SVD), (3) with > 70% stenosis in two major coronary blood vessels cases as ‘Double Vessel Disease’ (DVD) and, (4) with > 70% stenosis in three major coronary blood vessels as ‘Triple Vessel Disease’ (TVD). Based on the phenotypic severity, the CAD cases were also categorized into three broad conditions; (1) those with characteristic symptoms of stable or unstable ‘angina’, (2) with symptoms of ‘Acute Coronary Syndrome’ (ACS) and, (3) with reported ‘Myocardial Infarction’ (MI). However, we could not retrieve relevant information for categorizing CAD cases into the above subcategories for some of the case samples hence there was a difference in the total number of CAD cases used for anatomical and phenotypic severity categories when compared to the sample of pooled CAD cases.

### Statistical methods

The pooled sample of CAD case and control groups and each of the anatomical and phenotypic severity categories were subjected to pertinent statistical analyses. The data pruning, logistic regression analysis with and without covariates (age and sex) were done for 61 SNPs using PLINK software version1.07. After data pruning, only 45 of the 61 SNPs that showed minor allele frequency > 1% and conformed to Hardy–Weinberg Equilibrium (HWE) were qualified for further statistical analyses. The p-value for the association to be significant is set at 0.05 for a single SNP and after Bonferroni correction for multiple testing (*p* = α/m, where α = 0.05 and m = number of hypotheses or SNPs). Inter group (anatomical and phenotypic) differences were also analysed using PLINK after appropriate categorization of data into these groups as mentioned above. The post-hoc power of the study was calculated using G* power software (vs 3.1.9.7). The ‘SNPassoc’ package of R-PROGRAM was used for genotypic association analyses by considering different genetic models- co-dominant, over-dominant, dominant, recessive and log-additive. The model with significant *p* value and lowest AIC (Akaike Information Criterion) was selected as the best fit for the respective SNP. Linkage disequilibrium and haplotype analyses were done using HAPLOVIEW (version4.2.). The cumulative risk scores were obtained using SPSS (version 25, IBM) software.

Pair-wise SNP-SNP interactions among the 61 SNPs of the present study and between each of these SNPs and the SNPs of 11q23.3 and 9p21.3 regions earlier studied by us were analyzed using PLINK software version1.07. The multiple SNP interaction analysis was done with the help of non-parametric approach by GMDR (version 0.9), where a tenfold cross-validation with 2, 3, 4 and 5 way interactions were used to detect the gene–gene interactions. Based on the testing balance accuracy and minimal prediction error, the significant interactions were selected. The cumulative risk score for each individual was calculated based on the number of significant SNPs and to determine the predictive potential of the risk variants for CAD, the logistic regression analysis of the risk categories was performed and the receiver operating characteristic (ROC) curve was constructed using SPSS (version 25) and the area under curve (AUC) that reflects the prognostic potential of the risk variants determined.

## Results

### Allelic association of the SNPs with CAD in the pooled cohort

The minor allele and genotype frequencies of 61 SNPs for the CAD cases and controls were given in the Additional file 3: Table S2. However, after the data pruning using PLINK software, 16 of the 61 SNPs were excluded either because of minor allele frequency < 0.01 or deviation from Hardy–Weinberg equilibrium and the remaining 45 SNPs were subjected to further analyses. The logistic regression analyses of the allelic data in the pooled cohort revealed that five of the 45 SNPs were significantly associated with CAD (*p* < 0.05) four of which remained highly significant even after Bonferroni corrections for multiple testing (Table 1). The odds ratios of associated SNPs indicated that the minor alleles of the following SNPs; ***G****-rs10455872, ****C****-rs6725887, ****T****-rs782590, ****T****-rs173539* significantly increased the risk for CAD with highly elevated frequencies among CAD cases. The fifth SNP (*rs9818870*) was marginally significant and protective in nature. An increasing value of the odds ratios of the risk associated SNPs was observed in the order of *rs782590* (*SMEK1*)*, rs173539* (*HERPUD1-CETP*)*, rs6725887* (*WDR12*) and *rs10455872* (*LPA*) which were also implicated in the increasing severity of the CAD. However, the genes in which the associated SNPs are located appear to have diverse functions suggesting the role of these SNP variants in contributing to the genetic heterogeneity of CAD. These genes encode for proteins such as serine/threonine protein phosphatase, Homocystein inducible endoplasmic reticulum stress inducible ubiquitin like domain member 1-cholesteryl ester transfer protein, WD repeat containing domain 12 (ribosome biogenesis protein) and, lipoprotein-A respectively. The only SNP (*rs9818870*) that was not significant after corrections for multiple testing showed protective nature of its association with CAD with higher frequency in controls. The association of all the five SNPs were significant even after adjusting for age and sex.

**Table 1:** Allelic association of significant SNPs with coronary artery disease

SNP ID	Chr/gene location	Alleles	Minor allele frequency (MAF)		χ^2^	Unadjusted		Adjusted for age and sex	
		Minor/major	Cases (N = 350)	Controls (N = 480)		OR (CI 95%)	*p* value	OR (CI 95%)	*p* value
rs10455872*	6/ *LPA*	G/A	0.210	0.007	194.8	35.9 (16.7–77.2)	2.83e^−44^	60.0 (26.7–134)	3.54e^−23^
rs6725887*	2/*WDR12*	C/T	0.416	0.020	93.5	8.36 (5.16–13.8)	4.04e^−22^	8.58 (5.09–14.5)	7.22e^−16^
rs782590*	2/*SMEK1*	T/C	0.304	0.222	14.2	1.53 (1.23–1.92)	0.0002	1.51 (1.21–1.88)	0.0003
rs173539*	16/*HERPUD1-CETP*	T/C	0.037	0.011	9.8	3.54 (1.53–8.21)	0.002	2.86 (1.27–6.45)	0.011
rs9818870	3/*MRAS*	T/C	0.080	0.110	4.2	0.70 (0.50–0.99)	0.041	0.69 (0.49–0.97)	0.032

LPA, lipoprtein A; WDR12, WD repeat domian12; SMEK1, serine/threonine protein phosphatase/suppressor of mek1; HERPUD1-CETP, homocysteine-inducible, endoplasmic reticulum stress-inducible, ubiquitin-like domain member 1-cholesteryl ester transfer protein; MRAS, muscle RAS oncogene homolog

*SNP significant after correction for multiple testing

In order to check the internal consistency of our results and to validate the association of SNPs that were significant in the pooled cohort, we analysed 30%, 50% and 70% random subsets of our case and control cohorts and observed quite similar pattern of allelic association when compared to the total cohort for the four highly significant and risk conferring SNPs even after correction for multiple testing excepting for *rs782590* in the 30% subset (Additional file 4: Table S3). In addition, the post-hoc power of the study was calculated using G power for the four significant risk conferring SNPs with respect to the putative odds observed, taking the log of odds ratio as the effect size and the total sample size 830. The SNPs rs6725887, rs782590 and rs173539 with significant odds ratios of 8.58, 1.51 and 2.86 (Table 1) yielded statistical power (1 − β error probability) of 100%, 99.9% and 100% respectively. The SNP rs10455872 with odds ratio 60.0 was out of range with respect to log of odds (1.778) since the range of effect size considered in the G*power software is 0–0.999. High odds observed for the SNP could be attributed to the highly elevated minor allele frequency among cases and deviation from HWE which might suggest positive selection of the allele in the disease group. Hence, we used an online post-hoc power calculator (https://clincalc.com/stats/Power.aspx) for computing the power using proportion of minor allele and the sample size of both cases and controls which revealed power as 100%. These results suggested internal consistency and replicability of the results of association of the significant SNPs in our study.

### Allelic association of the SNPs with the anatomical categories of CAD

The results of logistic regression analyses of the 45 SNPs suggested altogether nine SNPs to be significantly associated (*p* < 0.05) with at least one of the four anatomical categories. The minor allele frequencies of the associated SNPs along with respective odds ratios were presented in Table 2. The four SNPs which were shown to increase the risk for CAD under allelic association analysis of the total sample were all found to be significantly associated with the SVD and DVD categories. Excepting *rs782590*, remaining three SNPs were associated significantly with increased risk for TVD. Two SNPs (*rs10455872, rs6725887*) remained significant in all categories after correction for multiple testing whereas, *rs782590* and *rs173539* were significant only in the DVD and SVD categories respectively. In addition to the association of these four SNPs with anatomical categories of CAD, the minor allele frequency of *rs247617* in the *CETP* gene was found to be significantly elevated in SVD (0.333) and TVD (0.356) categories of CAD compared to controls (0.259) with p values 0.036 and 0.019 respectively. While two additional SNPs; *rs2107595* (of *TWIST1* gene) and *rs3127599* (of *LPAL2* gene) were shown to confer protection (Odds Ratio (OR): 0.57) and risk (OR: 1.46) respectively to SVD, both the additional SNPs associated with TVD (*rs9940128- FTO* gene and, *rs1083096- MTNR1B* gene) were shown to reduce the risk. The genes to which these additional SNPs belonged were found to have diverse functional roles indicating possible genetic heterogeneity in the manifestation of different anatomical categories of CAD.

**Table 2:** Association of significant SNPs with the anatomical categories of coronary artery disease

SNP (minor/major allele)	MAF in controls N = 480	Insignificant (n = 81)			Single vessel disease (n = 98)			Double vessel disease (n = 70)			Triple vessel disease (n = 66)		
		MAF	OR (95%CI)	*p* value	MAF	OR (95%CI)	*p* value	MAF	OR (95%CI)	*p* value	MAF	OR (95%CI)	*p* value
rs10455872 (G/A)	0.007	0.234	41.2 (17.9–94.4)	*1.22e^**−41**^	0.177	29.1 (12.6–66.8)	*1.22e^**−30**^	0.164	26.5 (11.1–63.3)	*6.20e^**−26**^	0.206	35.0 (14.8–82.7)	*4.38e^**−34**^
rs6725887 (C/T)	0.020	0.198	12.0 (6.63–21.9)	*2.03e^**−23**^	0.119	6.58 (3.51–12.3)	*3.09e^**−11**^	0.123	6.87 (3.48–13.6)	*2.56e^**−10**^	0.138	7.86 (4.01–15.4)	*3.53e^**−12**^
rs173539 (T/C)					0.054	5.19 (1.85–14.6)	*0.0005	0.042	3.97 (1.17–13.4)	**0.017**	0.048	4.56 (1.34–15.5)	**0.008**
rs782590 (T/C)					0.295	1.47 (1.03–2.07)	**0.030**	0.377	2.12 (1.45–3.09)	*7.16e^**−5**^			
rs247617 (A/C)	0.259				0.333	1.43 (1.02–1.99)	**0.036**				0.356	1.58 (1.07–2.32)	**0.019**
rs2107595 (T/C)	0.325				0.216	0.57 (0.39–0.83)	*0.003						
rs3127599 (A/G)	0.205				0.274	1.46 (1.03–2.09)	**0.035**						
rs9940128 (A/G)	0.440										0.326	0.61 (0.42–0.90)	**0.013**
rs1083096 (C/G)	0.468										0.364	0.65 (0.44–0.95)	**0.024**

Bold indicates significant *p* value (of the odds ratio)

*p value significant after correction for multiple testing

### Allelic association of the SNPs with the phenotypic severity categories of CAD

The results of allelic association of SNPs with three phenotypic severity categories of CAD suggested eight SNPs to be significantly (*p* < 0.05) associated with at least one of the three phenotypic categories (Table 3). The four SNPs that were significantly associated with CAD in the pooled sample and with the anatomical categories showed significant association with phenotypic categories as well. While the *rs10455872* and *rs782590* were associated significantly with all three anatomical categories of CAD, the association of *rs6725887* was observed in angina and ACS categories and, *rs173539* in ACS and MI categories only. Additionally, significant increase in risk for angina, ACS and MI categories was observed with reference to *rs174546, rs1122608* and *rs4846922* respectively. The *rs7767084* was the only SNP shown to reduce the risk for angina. Except for the four common SNPs which showed significant allelic association in the pooled cohort, anatomical, and phenotypic categories, the additional SNPs appeared in the phenotypic categories were completely different from those observed in the anatomical categories of CAD. These results might help in identifying the genetic determinants of the clinical heterogeneity of CAD. The additional risk conferring SNPs of angina, ACS and MI categories were found to be located in *FADS1, SMARCA4* and *GALNT2* genes respectively and encode for proteins with diverse functional roles like lipid metabolism, chromatin remodelling and oligosaccharide biosynthesis suggesting that the unique set of SNPs associated with the phenotypic severity categories of CAD belonged to diverse functional pathways. However, a specifically designed study with relatively larger samples for subcategories of CAD might be required in order to confirm these findings.

**Table 3:** Association of significant SNPs with the phenotypic categories of coronary artery disease

SNP (minor/major allele)	MAF in controls N = 480	Angina(n = 73)			Acute coronary syndrome (n = 159)			Myocardial infarction (n = 75)		
		MAF	OR (95%CI)	*p* value	MAF	OR (95%CI)	*p* value	MAF	OR (95%CI)	*p* value
rs10455872 (G/A)	0.007	0.218	37.6 (16.2–87.4)	*1.80e^**−37**^	0.240	42.5 (19.3–93.7)	*3.06e^**−47**^	0.068	9.90 (3.71–26.5)	*2.63e^**−08**^
rs782590 (T/C)	0.222	0.340	1.81 (1.24–2.64)	*0.002	0.290	1.43 (1.07–1.91)	**0.014**	0.311	1.58 (1.08–2.31)	**0.017**
rs6725887 (C/T)	0.020	0.185	11.1 (5.98–20.6)	*2.44e^**−20**^	0.172	10.2 (5.91–17.4)	*1.60e^**−23**^			
rs173539 (T/C)	0.011				0.033	3.09 (1.15–8.33)	**0.019**	0.092	9.26 (3.26–26.3)	*5.28e^**−07**^
rs174546 (T/C)	0.095	0.151	1.68 (1.02–2.77)	**0.041**						
rs7767084 (C/T)	0.307	0.226	0.66 (0.44–0.99)	**0.046**						
rs1122608 (T/G)	0.239				0.297	1.34 (1.01–1.79)	**0.040**			
rs4846922 (T/C)	0.379							0.542	1.93 (1.36–2.75)	*0.0002

Bold indicates significant p value (of the odds ratio)

*p value significant after correction for multiple testing

### Genotypic association of the SNPs with CAD

The results of logistic regression analysis of the genotypes of five significant SNPs (Table 4) suggested that the SNP *rs10455872* with highly significant allelic association showed 48.5-fold (95%CI 22.3–105) increased risk for CAD in the presence of heterozygote *AG* (*p* < 0.0001) under co-dominant model. This result might suggest significant functional role of the SNP in contributing to CAD pathology. Additionally, both the heterozygous and homozygous variant genotypes of *rs6725887* and *rs782590* showed significant risk for CAD under log additive model with highly significant p values. However, the other SNP *rs173539* which showed significant allelic association failed to show genotypic association with CAD. Protective role conferred by the SNP *rs9818870* at the allelic association level did not turn out to be significant at the genotypic level. The lack of association of these SNPs at the genotype level could be because of the extremely low frequency of variant allele (*T*) among both cases and controls.

**Table 4:** Genotypic association of significant SNPs with coronary artery disease

SNP	Genotype	Frequency		OR(95% CI)	*p* value	Best model (p value)
		Cases n(%)	Controls n(%)			
rs10455872	AA AG GG	193 (57.96) 140 (42.04) 0 (0)	468(98.53) 7(1.47) 0(0)	Reference **48.5(22.3–105)** NA	**–** ** < 0.0001** NA	Co dominant (NA)
rs6725887	TT TC CC	251 (72.54) 89 (25.72) 6 (1.73)	455(95.99) 19(4.033) 0(0)	Reference **8.49(5.05–14.3)** **23.5(1.32–419)**	**–** ** < 0.0001** **0.032**	Log-additive (5.21e^−21^)
rs782590	CC CT TT	175 (50.72) 130 (37.68) 40 (11.59)	292(61.99) 149(31.63) 30(6.37)	Reference **1.45(1.08–1.97)** **2.22(1.34–3.70)**	**–** **0.014** **0.002**	Log-additive (0.0004323)
rs173539	CC CT TT	226 (93.77) 12 (4.98) 3 (1.24)	361(97.83) 8(2.17) 0(0)	Reference 2.40(0.96–5.95) 11.2(0.57–217)	**–** 0.060 0.111	Log-additive (0.011648)
rs9818870	CC CT TT	293 (85.17) 47 (13.66) 4 (1.16)	381(80.04) 85(17.86) 10(2.10)	Reference 0.72(0.49–1.06) 0.52(0.16–1.67)	**–** 0.095 0.273	Log-additive (0.2571)

Bold indicates significant *p* value/odds ratio

### Linkage disequilibrium (LD) and SNP-SNP interactions among the SNPs

The GWAS SNPs selected for analysis in the present study were traced to the sub chromosomal loci of 17 different chromosomes which indicated the presence of more than one SNP on one chromosome. The LD analysis identified 15 SNPs in 105 pair wise combinations (Additional file 1: Fig. S1, Figure legends). Overall, a disrupted LD pattern was seen with only 5 SNP pairs which reported *r*^2^ > 0.8 and none of the haplotypes were found to be significantly associated with CAD. On the other hand, neither pair wise nor the multiple SNP interaction analysis (generalized multifactor dimensionality reduction-GMDR) of these SNPs yielded any significant epistatic interactions associated with CAD among them.

### Cumulative risk score analysis for CAD associated SNP variants

**Fig. 1:**
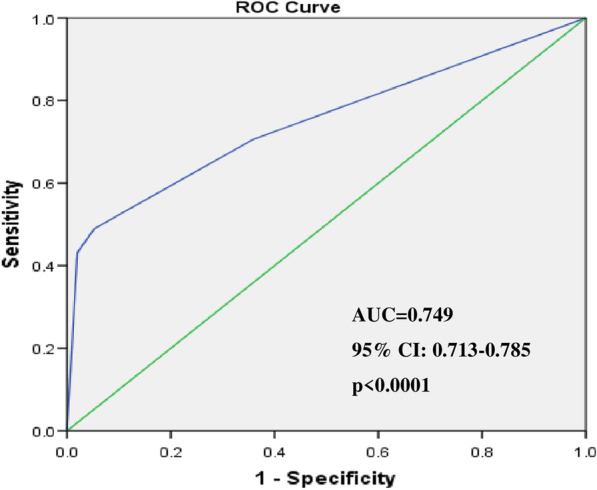
Receiver Operating Characteristic (ROC) curve indicating the area under curve (AUC) and the discriminative power of risk scores for five significant GWAS SNPs

The cumulative risk score analysis involved computation of weighted mean proportion of the risk alleles of the five significant SNPs by taking 2 for two risk alleles, 1 for one risk allele and 0 for no risk alleles with weights as relative log odds ratios of different SNPs. The cumulative risk score was obtained for each individual by multiplying with 5, the number of significantly associated SNPs. The individuals were grouped into 4 risk categories with increasing risk scores. Odds ratios and Z-scores were calculated by taking risk category 1 as the reference and the results were presented in Table 5. The frequency distribution of CAD cases and controls in different risk categories showed an increasing trend in the frequency of cases relative to controls specifically for the high-risk score categories 3 and 4 with risk scores in the range of 1.10–2.09 and 2.10–6.09 respectively. Furthermore, an increasing trend in the odds ratios and Z-scores was also apparent with increasing risk categories. The ROC curve (Fig. 1) yielded an area under curve (AUC) as 0.749 (95%CI 0.713–0.785) at *p* < 0.0001 which was statistically highly significant indicating possible predictive utility of the associated SNPs in CAD pathology.

**Table 5:** Cumulative risk score for CAD associated five significant GWAS SNPs

Risk category	Risk score	Cases (%)	Controls (%)	Odds ratio (95% CI)	Z-score	*p* value
1	0–0.59	29.45	64.17	Reference	**–**	**–**
2	0.60–1.09	21.57	30.55	**1.54 (1.07–2.21)**	2.337	**0.019**
3	1.10–2.09	5.83	3.3	**3.85 (1.90–7.81)**	3.742	**0.0002**
4	2.10–6.09	43.15	1.98	**47.54 (23.37–96.69)**	47.542	** < 0.0001**

Bold indicates significant *p* value/odds ratio

### Epistatic interaction of the 61 SNPs with the SNPs of 11q23.3 and 9p21.3 regions

The process of atherosclerosis is a result of disruption in lipid metabolism and cell proliferation pathways that was evident from significant association of few SNP variants at 11q23.3 and 9p21.3 loci respectively. While there were no significant pair wise interactions among the SNPs of the present study, it would be interesting to test if interaction of any of these SNPs with those in the 11q23.3 and/or 9p21.3 region play significant role in the manifestation of CAD. The pair wise interaction analysis suggested that the *rs7582720* of *WDR 12* gene on chromosome 2, albeit not individually associated with CAD, was found to be significantly conferring risk through epistatic interaction with the two SNPs (*rs6589566, rs1263163*) of 11q23.3 region which were found to be located in *ZPR1* gene and intergenic region of *APOA5-APOA4* genes respectively (Table 6).

**Table 6:** Significant SNP-SNP interactions between 61 SNPs and SNPs of 11q23.3, 9p21.3 loci associated with coronary artery disease

Chromosomal location	SNP Pair		Odds ratio	*p* value
	SNP1 (Chr 2)	SNP2 (11q23.3)		
Between Chr 2& 11q23.3SNPs	**rs7582720** (*WDR 12*)	**rs6589566**(*ZPR1*)	13.31	3.31e^−05^
		**rs1263163**(*APOA5-APOA4*)	8.530	7.09e^−05^
		rs10488699 (*BUD13*)	0.103	1.54e^−05^

Bold indicates risk SNP combinations

## Discussion

The case–control studies conducted so far among the Indian populations could help in identifying the CAD associated candidate genes which belonged to the most replicated GWAS loci such as 9p21.3 and 11q23.3 [14–16]. However, given the complex nature of the disease and the large number of candidate genes associated with CAD worldwide, these studies were not adequate to characterize the genetic susceptibility profile of either the local or regional populations. Further, the Indian studies could validate only a few conventional polymorphisms of the genes located in these two chromosomal regions. Given the distinct nature of association of the SNP variants of 11q23.3 and 9p21.3 regions with CAD in our earlier studies of the same cohort involving relatively large number of SNPs [7–9], the association of variants from the other genomic loci with functional significance to CAD were evaluated in the present study and observed unique pattern of association of the SNPs in this Southern Indian population of Hyderabad as compared to the other populations from India and elsewhere [1, 14].

We found significant association of four of the 45 SNPs in elevating risk for CAD of which *rs10455872(A* > *G)* was highly significant with relatively much higher value of odds ratio (Table 1). This SNP was also consistently associated with high risk in different phenotype specific cohorts based on anatomical and clinical categories including the TVD, ACS and MI. Significant association of this SNP was also validated before in other studies wherein it was shown to be associated with acute myocardial infarction [17], coronary lesions in Brazilian patients submitted to coronary angiography [18] and calcific aortic valve disease in Bulgaria [19]. The Lipoprotein(a) [Lp(a)] is a low-density lipoprotein (*LDL*) bound to apo(a), a plasma apolipoprotein [20, 21] which was shown to be involved in lipoprotein metabolism. *Lp(a)* was considered as a candidate gene of CAD and high concentration of this protein was found to be a documented risk factor [22, 23]. Even though Indians were known to have a unique pattern of dyslipidemia usually characterized by low levels of LDL cholesterol with predominantly atherogenic and small-dense LDLs [24, 25], a study from the north Indian population did not show association of this SNP with CAD [26]. However, a recent study revealed that the variant *G* allele of the SNP *rs10455872* was associated with increased Lp(a) protein levels and aortic valve calcification [27]. This SNP was found to be located in the intron 25 of lipoprotein *Lp(a)* gene on chromosome6 (Additional file 2: Table S1). Since the SNP was found to be significantly associated with CAD in our study, we tried to understand the effect of variant allele (G) on the protein structure or function through in silico analysis. Alternative Splice Site Predictor (ASSP) analysis indicated that the presence of G allele might result in the formation of cryptic donor splice site and/or alternative isoform (http://wangcomputing.com/assp/). Hence, it could be suggested that the presence of variant allele *G* of *rs10455872* in the intronic region of the *Lp(a)* gene might affect splicing and rate of gene expression resulting in defective lipoprotein metabolism and subsequent development of CAD.

The second highly significant SNP associated with CAD was *rs6725887* with an odds ratio of 8.58. This SNP was found to be located in the intronic region of *WDR12* (WD repeat domain12) gene on chromosome2 which encodes for a ribosome biogenesis protein. Other studies did not find association of this SNP with CAD risk factors such as hypertension, lipid traits and atherosclerosis [28, 29]. Further, meta-analysis of this SNP did not show significant association with CAD in the sub-Asian populations [30]. In contrast, the *rs9818870* located in the *MRAS* (muscle RAS oncogene homolog) gene on chromosome3 (3q22.3), which appeared to decrease the risk for CAD in the present study, was found to be the most replicated SNP identified in GWAS susceptible CAD loci [16]. Despite the fact that there were no other validating studies to show the association of SNPs *rs782590* and *rs173539* with CAD, the present study found significant association of these SNPs with increased risk for CAD. Even though the functional role of these SNPs was not characterized yet, it could be suggested that the proteins coded by the genes in which these SNPs are located might have significant effect in the process of development of CAD.

The four significant CAD associated risk SNPs in the pooled cohort are located on different genes whose protein products have primary role in lipid metabolism (*LP(a)*) and other following events such as cell division and proliferation (*WDR* *12, SMEK1, HERPUD1-CETP*) in atherosclerosis and development of CAD. The distinct patterns of association of the SNPs with anatomical and phenotypic categories of CAD suggested the possible role of various SNPs in contributing to the genetic and etiologic heterogeneity of CAD, albeit relatively larger sample size for the subcategories of CAD would have provided sufficient statistical power and greater degree of confidence in the inference. Furthermore, the pair wise SNP associations observed between one of the SNPs of the present study (*rs7582720* of *WDR* *12* gene on chromosome2) and three SNPs of 11q23.3 region (belonging to *ZPR1*, *APOA5-APOA4* and, *BUD13* genes) also suggested unique epistatic interactions of SNPs in CAD pathology. Variants of 11q23.3 chromosomal region which showed epistatic interactions in the present study are found to be located in apolipoprotein encoding or regulating genes hence might be involved in defective cholesterol homeostasis thereby resulting in increased levels of oxidised low-density lipoproteins (LDLs). Subsequently, the *WDR* *12* gene on chromosome 2 harbouring the SNP *rs7582720* encode for a ribosome biogenesis protein involved in cell proliferation which might play a role in the later event of atherosclerosis. Therefore, the complex nature of CAD phenotype might be suggested to be the outcome of interactions of different genomic loci. However, the in vitro studies on the expression levels of these genes might help in validating the hypothesis.

Although candidate genes and GWAS loci for CAD were found to be replicated in the present study and in few other population-based studies, the SNP variants showing association were found to be different for different populations [2, 31] suggesting the population specific association patterns of GWAS or candidate gene variants with CAD development and/or progression. Interestingly, the current study which attempted to comprehensively explore the pattern of association of variants by considering large number of SNPs across the genomic regions also yielded a different set of SNPs in the population of Hyderabad, India which was evident by the association of *rs10455872* of *LP(A)* gene that showed profound risk for CAD in this regional Indian population. Perhaps, this can be a new and significant observation of our study. Earlier studies on the Indian population did not find the association of this SNP with CAD and although the GWAS for CAD identified *LP(A)* as one of the candidate genes, it revealed the association of a different SNP (*rs3798220*) of this gene with CAD. The other four SNPs associated with CAD were also unique for the current study population which were either not studied or not shown to be associated before in the Indian population. Overall, the present study observed that the variants associated with CAD in the population of Hyderabad are unique and with high discriminative power suggesting the possibility of their utility as predictors of the risk for CAD.

## Conclusions

The present study could help in identifying unique set of SNPs significantly associated with CAD specifically in the Southern Indian population of Hyderabad. The highly significant odds ratios and their magnitudes observed in the present study suggested that the SNPs rs10455872 and rs6725887 might have independent role, but not in additive fashion, as disease causing variants in CAD which demands further studies and analyses of these SNPs in different populations and by excluding the confounding nature of the effects of associated parameters such as lipid profiles to confirm its independent effect. The distinct pattern of association of different SNPs observed with respect to anatomic and phenotypic categories of CAD also suggested genetic and etiologic heterogeneity of CAD and warrants that CAD patients may be screened for these SNPs in order to explore the genetic susceptibility profile behind the clinical heterogeneity of CAD. However, given the relatively smaller sample sizes for the sub-phenotypes, the present study can be considered exploratory to establish a population wide SNP association pattern for CAD by using specifically designed large scale studies among different populations of India, both local and regional.

## Supplementary Information

**Additional file 1. Figure S1: **Linkage disequilibrium plot of GWAS SNPs. In the LD plot, each square/block displays the magnitude of LD in terms of D’ value for a pair of markers. The strength of LD between markers is indicated by the colour intensity of the box. LD ranges from 0–100 which is denoted as D’ (0-1). D’ value < 0.30 indicates low LD score, D’ 0.50–0.70 indicates moderate LD and, D’ > 0.70 indicates high LD scores between the markers. Red colour boxes indicate high LD scores between markers and, the boxes in a block represent haplotype combinations.

**Additional file 2. Table S1: **Chromosomal, gene locations and functions of the 61 SNPs selected for the present study in Coronary Artery Disease

**Additional file 3. Table S2: **Minor allele and Genotype frequencies of 61 SNPs in CAD cases and controls

**Additional file 4. Table S3: **Subset analysis (30%, 50%, 70%) of CAD cases and controls to check for the internal consistency and replicability of significant SNPs.

## Data Availability

The datasets used and/or analyzed during the current study available from the corresponding author on reasonable request.

## Abbreviations

GWAS: Genome wide association studies
SNPs: Single nucleotide polymorphisms
CAD: Coronary artery disease
NHGRI: National Human Genome Research Institute
ROC: Receiver operating characteristics
AUC: Area under curve
DNA: Deoxyribonucleic acid
SVD: Single vessel disease
DVD: Double vessel disease
TVD: Triple vessel disease
ACS: Acute coronary syndrome
MI: Myocardial infarction
HWE: Hardy Weinberg equilibrium
MAF: Minor allele frequency
OR: Odds ratio
CI: Confidence interval
LD: Linkage disequilibrium
GMDR: Generalized multidimensionality reduction
SMEK1: Serine/threonine protein phosphatase/suppressor of mek1
HERPUD1: Homocysteine-inducible, endoplasmic reticulum stress-inducible, ubiquitin-like domain member 1
CETP: Cholesteryl ester transfer protein
WDR12: WD repeat domian12
LPA: Lipoprtein A
ZPR1: Zinc finger protein 1
APOA5-APOA4: Apolipoprotein A5-apolipoprotein A4
BUD13: Bud homolog 13
MRAS: Muscle RAS oncogene homolog
LDL: Low density lipoprotein
