# Supplementary figures and images for: Analysis of 61 SNPs from the CAD specific genomic loci reveals unique set of SNPs as significant markers in the Southern Indian population of Hyderabad
Source: BMC Cardiovasc Disord. 2022 Apr 5;22:148. doi: 10.1186/s12872-022-02562-4 (PMC8981708; doi:10.1186/s12872-022-02562-4)

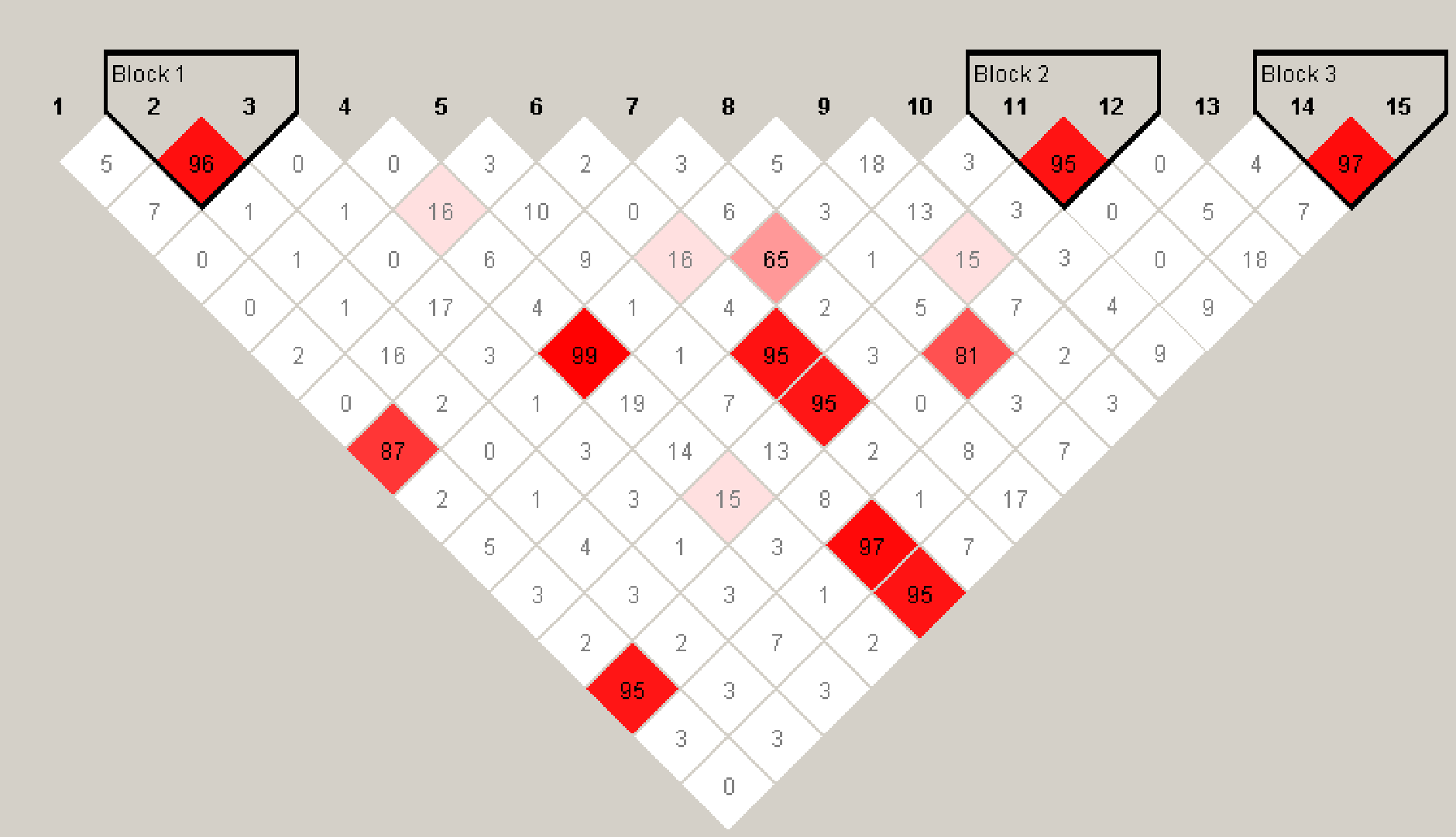

Supplement: Supplementary file 1 — Additional file 1. Figure S1: Linkage disequilibrium plot of GWAS SNPs. In the LD plot, each square/block displays the magnitude of LD in terms of D’ value for a pair of markers. The strength of LD between markers is indicated by the colour intensity of the box. LD ranges from 0–100 which is denoted as D’ (0-1). D’ value < 0.30 indicates low LD score, D’ 0.50–0.70 indicates moderate LD and, D’ > 0.70 indicates high LD scores between the markers. Red colour boxes indicate high LD scores between markers and, the boxes in a block represent haplotype combinations. [file 12872_2022_2562_MOESM1_ESM.tif]
